# Variations in deep-sea microbial composition and assembly mechanisms under different culture strategies

**DOI:** 10.3389/fmicb.2026.1783610

**Published:** 2026-03-18

**Authors:** Libo Yu, Jianxing Sun, Yuguang Wang, Xixiang Tang, Hongjian Tan, Hongbo Zhou, Haina Cheng, Zhu Chen

**Affiliations:** 1 Key Laboratory of Marine Genetic Resources, Third Institute of Oceanography, Ministry of Natural Resources, Xiamen, China; 2 School of Minerals Processing and Bioengineering, Central South University, Changsha, Hunan, China; 3 Department of Laboratory Medicine, Affiliated Nanhua Hospital, University of South China, Hengyang, China; 4 Key Laboratory of Biohydrometallurgy of Ministry of Education, Changsha, China

**Keywords:** co-occurrence pattern, community assembly, deep-sea microorganisms, high-pressure cultivation, marine sediments

## Abstract

Deep-sea sediments harbor diverse microbial resources with immense biotechnological potential. Cultivating of these microorganisms is essential for studying their physicochemical properties, ecological functions, and resource development but remains a significant challenge. This study investigated the effects of different cultivation conditions on microbial diversity, community composition, coexistence patterns, and assembly processes from Yap Trench sediments (4,159–6,682 m depth). Results indicated that 44–55% of the microbial diversity was captured from the original sediments under high-pressure cultivation conditions, significantly higher than the 6–18% obtained under atmospheric pressure. Communities cultivated under high-pressure oligotrophic conditions closely resembled *in situ* microbiota, forming complex co-occurrence networks with high modularity and predominantly positive interactions, indicative of cooperative relationships enhancing community stability. However, serial subculturing and atmospheric cultivation favored pressure-tolerant groups like *Proteobacteria*. The deterministic processes (particularly heterogeneous selection) of community assembly were enhanced under high-pressure conditions, despite stochastic processes remaining the dominant mechanism. These findings highlight the adaptive responses of deep-sea microbial communities to extreme environmental pressures and underscore the importance of simulating high-pressure oligotrophic conditions for enriching diverse microbial groups. This study advances our understanding of deep-sea microbiology and provides a framework for exploring microbial functions and resources in extreme environments.

## Introduction

Marine sediments, covering more than two-thirds of Earth's surface, represent the largest benthic ecosystem and a reservoir for vast microbial diversity (Teske and Sørensen, 2008). It is estimated that these sediments harbor microbial biomass estimated at 2.9–35.5 × 10^29^ cells, accounting for 55–86% of Earth's prokaryotic biomass and 27–33% of Earth's living biomass (Kallmeyer et al., 2012; Whitman et al., 1998). Microorganisms in marine sediments drive fundamental processes, including the degradation of organic matter, methane production, and sulfate removal from the ocean (D'Hondt et al., 2004). The deep sea is the largest marine ecosystem on Earth, accounting for approximately 75% of the ocean's volume and supports 62% of the global biosphere (Fang et al., 2010). Despite extreme conditions, including darkness, low temperatures, high pressures, and oligotrophy, deep-sea sediments harbor diverse and abundant microbial communities that possess specialized functional potentials and processes (i.e., microbial metabolic capabilities and biogeochemical functions) that are expressed under such conditions (Hiraoka et al., 2020; Peoples et al., 2019). However, despite their ecological significance, the physicochemical properties and ecological roles of microorganisms in deep-sea sediments remain poorly understood due to limitations in culturing techniques (Thrash, 2019; Zhang et al., 2019).

The emergence and innovation of high-throughput sequencing technology have revolutionized microbiology by enabling culture-independent genomic analyses, providing unprecedented insights into microbial diversity and function (Forster, 2017; Gutlebena et al., 2017; Parks et al., 2018; Satam et al., 2023). Although culture-independent multi-omics can robustly infer metabolic potential (metagenomics) and *in situ* transcriptional activity (metatranscriptomics), they often remain limited in establishing direct physiological traits, growth parameters, and causal functional validation (e.g., substrate utilization ranges, pressure tolerance limits, growth kinetics, and interactions under controlled conditions) without cultivation-based experiments (Lewis et al., 2021; Liu et al., 2022b; Verhoeven et al., 2023). For example, the complete nitrification in *Nitrospira* (Daims et al., 2015), and the reversible TCA cycle in *Thermosulfidibacter takaii* (Nunoura et al., 2018) were discovered only through pure culture experiments. Although cultivation is essential for mechanistic validation, the majority of environmental microorganisms (over 99%) remain difficult to isolate using traditional laboratory conditions (Alain and Querellou, 2009; Rygaard et al., 2017; Yang et al., 2023). This challenge is amplified in deep-sea sediments, where near-freezing temperatures, low nutrient limitation, and high hydrostatic pressure impose strong physiological constraints and can reduce culturability upon sample retrieval and depressurization (Lloyd et al., 2018; Zhang et al., 2019). Consequently, conventional atmospheric-pressure incubations often enrich a limited subset of pressure-tolerant opportunists rather than microorganisms representative of the *in situ* community (Carvalho et al., 2024; Kogure et al., 1979), underscoring the need for cultivation strategies that better reproduce key deep-sea conditions, particularly hydrostatic pressure (Button et al., 1993; Rygaard et al., 2017; Simu and Hagström, 2004). In hadal trench environments, hydrostatic pressure and oligotrophy are two defining features, reproducing these constraints during incubation is expected to improve enrichment of *in situ*-representative microorganisms. High-pressure cultivation can mitigate depressurization-driven selection against piezophilic taxa, whereas oligotrophic media can reduce the dominance of copiotrophic opportunists favored by nutrient repletion. Together, combining high pressure with reduced nutrient supply provides a mechanistically motivated strategy to broaden the cultivable fraction of deep-sea microbial dark matter while retaining key features of the native community. These approaches not only provide a practical foundation for isolating representative taxa and experimentally validating their physiology and ecological functions, but also expand access to deep-sea microbial resources with potential biotechnological value (Huang et al., 2023b; Lewis et al., 2010).

In this study, we employed two different culture media to cultivate microorganisms from deep-sea sediment samples collected at depths of 4,159–6,682 meters in the Yap Trench, a hadal trench system located in the western Pacific Ocean near the Caroline Plate (south of Japan and east of the Philippine Sea), under both atmospheric and high-pressure conditions. We analyzed the microbial community composition under these cultivation conditions and compared it with the *in situ* microbiota. The objectives of this study were to: (i) investigate differences in microbial diversity and community composition under different cultivation conditions; (ii) explore how cultivation conditions influence microbial co-occurrence patterns and community assembly processes; and (iii) determine which cultivation conditions are more effective for recovering *in situ* microbial communities. This study provides insights into the dynamics and assembly of deep-sea microbial communities under varying cultivation conditions, offering new perspectives on cultivating unculturable microorganisms and exploring their functional potential.

## Materials and methods

### Sediments collection and samples selection

**Figure 1:**
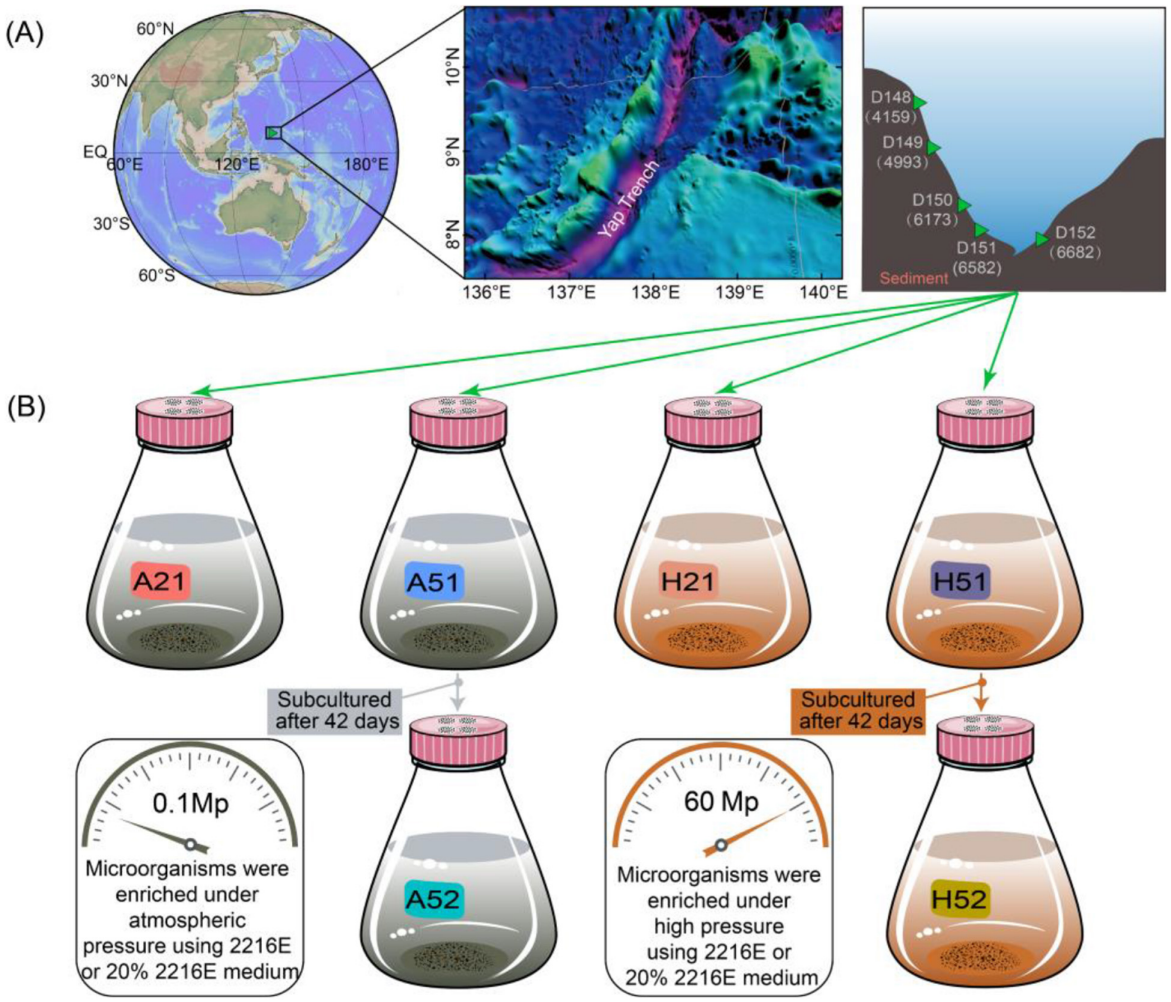
Schematic diagram of sampling sites and cultivation methods. **(A)** Sampling location of deep-sea sediments. **(B)** Schematic diagram of different cultivation strategies.

Yap Trench (8.02–8.06 °N, 137.52–137.84 °E) was selected for sampling sediments during the 38th (2017) Dayang cruises of China, sediments were sampled by manned submersible *Jiaolong* with push-core (diameter was 6 cm). Sediment cores were collected from the two opposing flanks of the Yap Trench axis, hereafter referred to as the eastern trench slope and western trench slope, these two slopes exhibit distinct morphological features, with the western slope being steeper and the eastern slope being deeper. Therefore, they may experience differences in sediment deposition and organic matter supply, providing a geological and ecological context for comparing *in situ* communities and their responses to cultivation under controlled pressure and nutrient regimes. Sampling locations are presented in Figure 1A, with sample details provided in Supplementary Table S1. Specifically, samples D148, D149, D150, and D151 were collected from the western slope of the Yap Trench, while sample D152 was from the trench's eastern slope. Upon recovery, sediments were immediately transferred into a pressure-retaining container to minimize depressurization prior to incubation. For subsequent enrichments, the preserved material was dispensed into culture vessels under sterile conditions and incubated either at 1 atm or maintained at high pressure (60 MPa) according to the experimental design. To minimize contamination, all subsampling was performed with sterile gloves and sterile, single-use tools; working surfaces were cleaned with 75% ethanol between samples. For each core, the outer layer 0.5–1 cm of sediment was removed aseptically, the two middle sections were selected for subsequent experiments and labeled as “a” and “b”, and material for downstream analyses and cultivation was taken from the core interior at the specified depth interval using sterile spatulas. Subsamples for DNA extraction were placed into sterile cryovials and flash-frozen in liquid nitrogen until laboratory processing.

### Enrichment culturing

Enrichment cultivation was initiated immediately after sample retrieval and onboard processing, in order to maintain sample freshness and microbial viability. To better approximate the conditions typical of deep-sea environments, we used full-strength 2216E (composition detailed in Supplementary Table S2) as the nutrient-rich condition and 20% (1/5-strength) 2216E as the low-nutrient (oligotrophic) condition to compare how different nutrient-supply regimes influence microbial enrichment outcomes. This diluted medium maintains seawater-like ionic composition while lowering organic carbon and nutrient availability, thereby increasing the likelihood of enriching slow-growing or oligotrophic taxa and improving the representativeness of enrichment communities relative to the *in situ* microbiota. Additionally, samples were cultured under two pressure environments: atmospheric pressure (1 atm, Group A) and high pressure (60 MPa, Group H). All cultures were incubated at 2 °C for 42 days, yielding enriched products labeled as A21, A51, H21, and H51. For cultures using 1/5 diluted 2216E medium, subculturing was performed by transferring 1% of the enriched product into fresh medium and continuing the enrichment for another 42 days, resulting in second-generation products labeled as A52 or H52 (as shown in Figure 1B).

The high-pressure enrichment procedure was as follows: (1) Subsampling: 1 g of sediment was placed in a sterile disposable polypropylene (plastic) syringe containing 10 mL of sterile medium and sealed with a sterile silicone stopper on the syringe needle. (2) Pre-cooling: A handheld high-pressure culturing chamber (see patent CN204022810U) was used for high-pressure incubations. To mimic the *in situ* temperature at the sampling sites (approximately 2 °C), all cultivation chambers were pre-cooled in a refrigerator set to 2 °C prior to incubation; this equilibration step was performed to stabilize the system at the target temperature and reduce temperature perturbation before cultivation. (3) Sample placement: The prepared syringe from step (1) was placed in the high-pressure chamber. (4) Pressurization: The pressure pump was used to reach the target pressure. Once the desired pressure was achieved, the water valve on the high-pressure chamber was closed, and the vent valve on the pressure pump was opened to release the water inlet slot. (5) Culturing: The high-pressure chamber was incubated at 2 °C (close to the *in situ* temperature at the sampling sites). During incubation, any pressure fluctuations due to temperature changes were corrected to maintain stability. (6) Sample collection: After incubation, the high-pressure chamber was removed, and the pressure relief valve was slowly opened to reduce the pressure to zero. The chamber was then opened for sample collection. The detailed schematic and configuration of the portable high-pressure microbial transfer and culturing device used for high-pressure cultivation are described in patent CN204022810U; its schematic diagram is shown in Supplementary Figure S1A, and a photograph of the device during cultivation is shown in Supplementary Figure S1B.

### DNA extraction, amplification, and sequencing

DNA extraction and amplification from the enriched products were conducted according to previously established protocols (Zhang et al., 2018). Briefly, 10 mL of each liquid enrichment culture was filtered through a 0.22 μm membrane filter (Merck Millipore, Billerica, MA, USA), and DNA was extracted from the cells retained on the filter using the MoBio PowerSoil DNA Isolation Kit (MoBio Laboratories, Inc.) following the manufacturer's instructions. The V4 region of the 16S rRNA gene was amplified using specific primers of 515F (5′-GTGCCAGCMGCCGCGGTAA-3′) and 806R (5′-GGACTACVSGGGTATCTAAT-3′). PCR amplification was performed under the following program: initial denaturation at 95 °C for 3 min; followed by 40 cycles of 95 °C for 5 s, 58 °C for 30 s, and 72 °C for 1 min. Amplicons were purified using the universal DNA Purification Kit (DP214-02, Tiangen, Beijing, China). Purified DNA were quantified using using a Nanodrop ND-1000 spectrophotometer (Nano-100, Aosheng Instrument Co Ltd.). Three technical replicates were run for each sample, and then the three replicated products were combined for sequencing. S Sequencing was performed using the MiSeq platform (Illumina Inc., San Diego, CA, USA) using paired-end 2 × 250 bp (PE250) sequencing at Guangzhou Gidio Biotechnology Co. Ltd.

### Quantitative PCR

The qPCR procedure followed methods previously described (Sun et al., 2023). Briefly, the quantitative PCR (qPCR) was used to quantify 16S rRNA gene copy numbers during enrichment. qPCR targeted the biological 16S rRNA gene V4 region using primers 515F and 806R (the same primer pair used for the initial V4 amplicon PCR). The qPCR was performed using a SYBR Green master mix (BioTeke, Beijing, China) on a real-time PCR system (Applied Biosystems, Foster City, CA, USA) under the following cycling program: 95 °C for 3 min; 40 cycles of 95 °C for 5 s and 58 °C for 30 s. Each DNA sample was quantified in triplicate technical qPCR reactions.

### Raw data processing, bioinformatics, and statistical analysis

The raw high-throughput amplicon data were processed using an in-house integrated pipeline as described in previous studies (Feng et al., 2017; Sun et al., 2024; Xiao et al., 2025). Briefly, sequencing reads were processed in the QIIME2 platform (Amplicon Distribution 2024.5). Adaptors and primers were trimmed using Cutadapt. Reads were quality-filtered (mean Q ≥ 20; length ≥ 200 bp) and paired-end reads were merged. Sequences were dereplicated and clustered into operational taxonomic units (OTUs) at 97% identity threshold using the UPARSE pipeline. To reduce bias from uneven sequencing depth, the OTU table was rarefied to 36,669 reads per sample. Representative OTU sequences were taxonomically classified using the SILVA database (version 138).

Alpha diversity was assessed to compare the diversity of microorganisms under different culture strategies, with richness (i.e., OTU number) and the Shannon index calculated using the “vegan” package in R. The overall differences in microbial beta diversity were visualized using principal coordinate analysis (PCoA) using the “vegan” package in R software, based on the Bray–Curtis dissimilarity. Canonical correspondence analysis (CCA) was also performed using the “vegan” package to reveal relationships between microbial community heterogeneity and culture conditions. All statistical analyses were conducted in R (version 4.0.1).

To examine community assembly mechanisms under different cultivation conditions, the null model was employed to quantify the relative contributions of deterministic and stochastic processes in shaping microbial communities based on the β-nearest taxon index (βNTI) and RC_bray_ values (Stegen et al., 2012, 2013). Co-occurrence networks were constructed across different culture conditions based on Spearman correlations. To enhance network visualization, only OTUs present in more than 50% of samples were included. Significant correlations were identified with absolute correlation coefficients (|*r*|) > 0.7 and *p*-values < 0.05. False discovery rate (FDR) adjustments were applied to the *p*-values using the Benjamini-Hochberg (BH) method to minimize false positives (Benjamini and Hochberg, 1995). Additionally, topological parameters, including nodes, links, average degree (*avgK*), modularity, and average clustering coefficient (*avgCC*), were calculated to characterize the network topology (Jiao et al., 2020; Zhang et al., 2021). All networks visualization and topological analysis were performed using Gephi software (version 0.9.2).

## Results

### Microbial alpha diversity changes under different cultivation conditions

**Figure 2:**
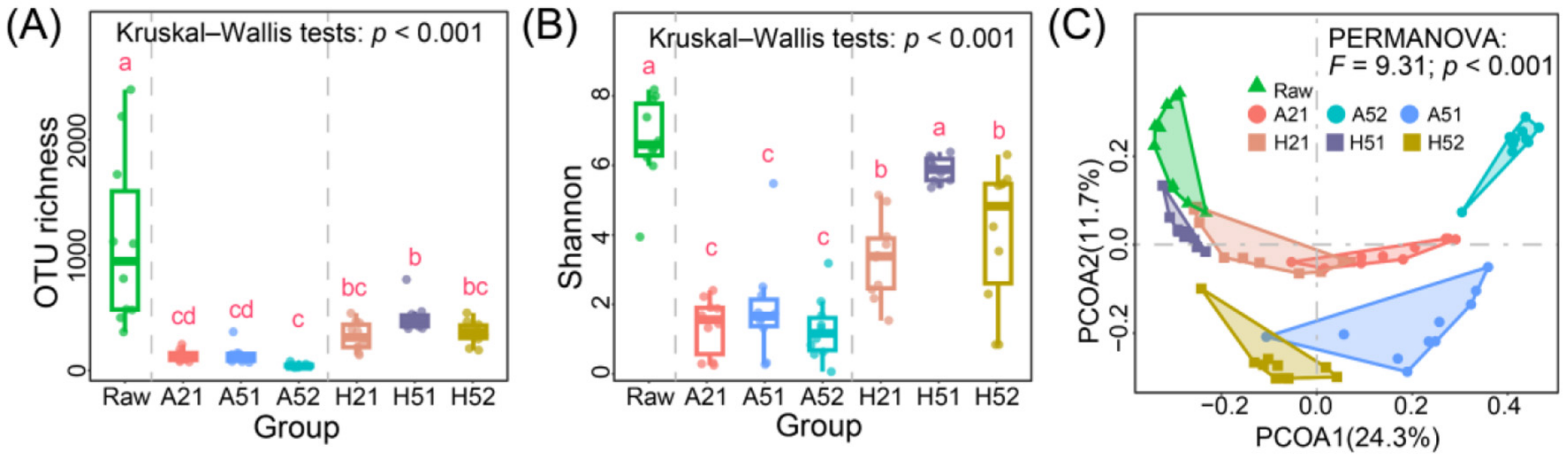
Microbial diversity and community structure analysis under different cultivation strategies. **(A,B)** Changes in microbial OTU richness and Shannon indices under different cultivation conditions, respectively. **(C)** Comparison of microbial community structures under different cultivation conditions based on PCoA analysis. OTU richness and Shannon diversity were compared among groups using Kruskal–Wallis's tests, followed by Dunn's post hoc pairwise comparisons with Benjamini–Hochberg correction. Community dissimilarity was tested using PERMANOVA based on Bray–Curtis distances (implemented in R via the adonis2 function).

After sequencing data preprocessing, rarefaction was performed at the sample with the lowest sequence count (36,669), yielding a total of 2,566,830 high-quality sequences (Supplementary Table S3). The rarefaction curve results showed that as sequencing depth increases, the number of captured OTUs reached a plateau (Supplementary Figure S2), indicating that the sequencing depth is sufficient to capture the OTU richness in these samples. The OTU richness values for *in situ* uncultured samples varied widely across sites (333–2,433), indicating very high OTU number with an average of 1,118 OTUs (Figure 2A). Alpha diversity indices (including OTU richness and the Shannon index) were compared among the seven groups using the Kruskal–Wallis's test, which indicated significant overall differences in diversity (*p* < 0.001). Subsequent pairwise comparisons (Dunn's post hoc tests with Benjamini–Hochberg correction) showed that enrichment products cultivated under high pressure exhibited significantly higher alpha diversity than those cultivated under atmospheric pressure, although their diversity remained lower than that of the original *in situ* habitat (Figures 2A, B). Notably, first-generation cultures under high-pressure and low-nutrient conditions (H51) had an average OTU richness value of 455, up to 41% of the *in situ* microbial OTU number (Figure 2). In contrast, atmospheric pressure cultivation without subculturing exhibited consistent microbial OTUs (averaging 135 for A21 and A51), while subculturing under low-nutrient atmospheric conditions (A52) resulted in the lowest diversity, averaging only 46, representing just 4% of the original microbial OTUs. Although both atmospheric and high-pressure cultivation resulted in significant diversity loss compared to the native habitat (Dunn's post hoc tests with Benjamini–Hochberg correction, *p* < 0.001, Supplementary Figure S3), high-pressure conditions consistently retained significantly higher microbial alpha diversity, particularly under oligotrophic conditions (H51 group). Atmospheric pressure cultivation captured only 6–18% of deep-sea microbial OTUs, while high-pressure conditions captured 44–55% (Supplementary Figure S3). In the original sediment samples, a total of 4,332 OTUs were identified, of which 3,078 (71.1%) were detected exclusively in the raw sediments and were not observed in any of the enrichment cultures in the laboratory (Supplementary Figure S4). High-pressure oligotrophic conditions totally yielded 1,611 OTUs, with 329 OTUs shared between high-pressure oligotrophic cultures and original samples, representing the highest number of shared OTUs. The Shannon index exhibited a trend consistent with OTU richness (Figure 2B). Microbial β-diversity analysis using PCoA revealed clear clustering of samples under different cultivation conditions (Figure 2C), exhibiting significant inter-group differences in microbial communities (*F* = 9.31, *p* < 0.001). Moreover, all groups showed significant community differences except for pre- and post-subcultured samples under atmospheric pressure and low-nutrient conditions (A51 vs. A52), which exhibited no significant differences in composition (Supplementary Table S4).

### Community composition changes under different culture conditions

**Figure 3:**
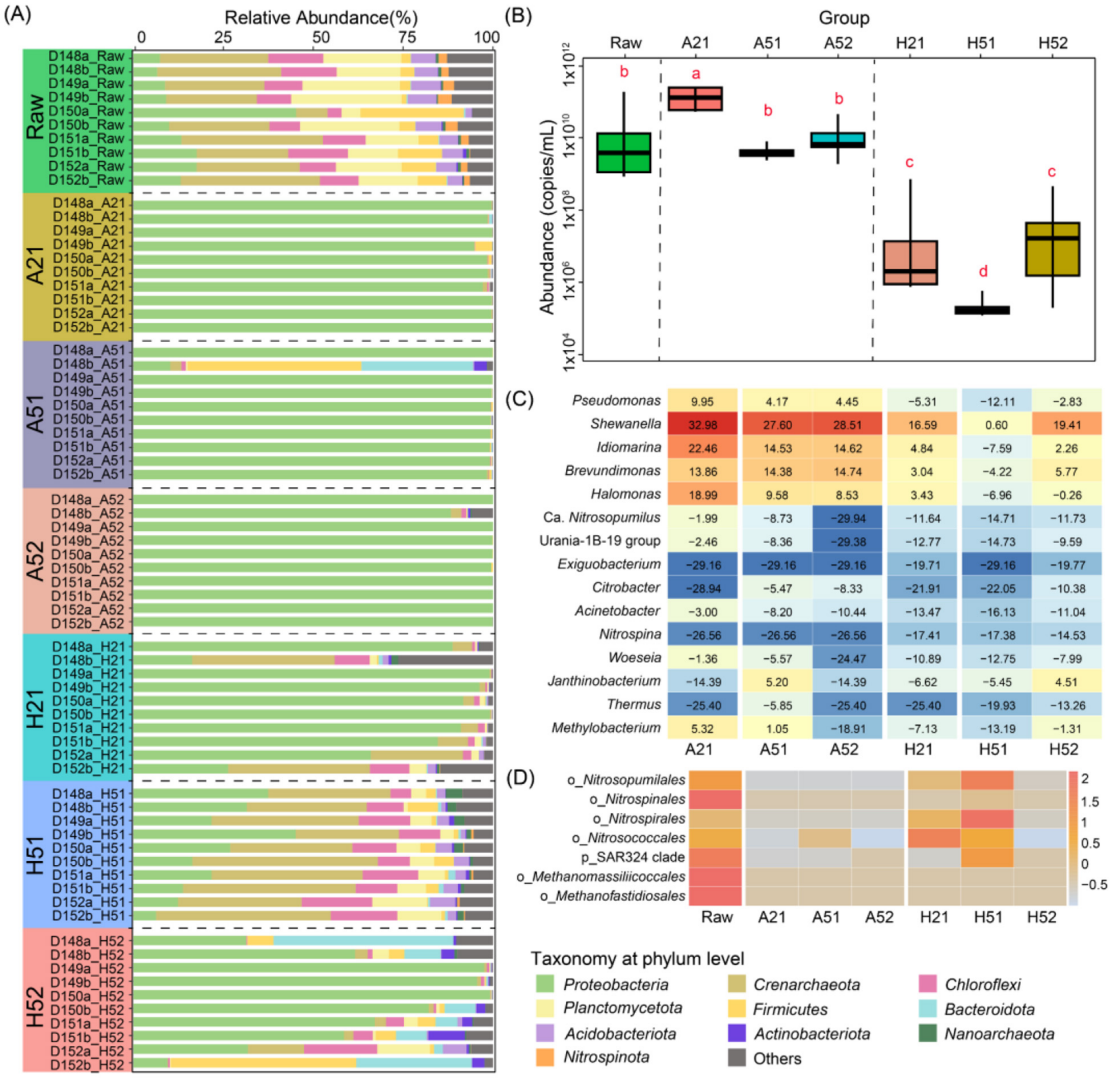
Changes in microbial community composition and abundance under different cultivation conditions. **(A)** Phylum-level composition of microbial communities under different cultivation conditions and changes in relative abundance (top 10 abundant phyla). **(B)** Changes in absolute abundance of microorganisms under different cultivation conditions (measured by copy number). **(C)** Comparison of microbial growth (fold increase) at the genus level under different cultivation conditions. **(D)** Changes in relative abundance of functional microorganisms under different cultivation conditions.

The dominant phyla in the raw Yap Trench sediments were *Crenarchaeota* (average 28.6%), *Planctomycetota* (average 19.3%), *Proteobacteria* (average 14.9%), *Chloroflexi* (average 11.3%), and *Firmicutes* (average 7.9%) at the phylum level (Figure 3A). Under atmospheric pressure cultivation conditions, the microbial community was overwhelmingly dominated by *Proteobacteria*, with an average relative abundance of 98.9% in all samples except D148b. In contrast, high-pressure nutrient-rich conditions (H21 group) enriched specific groups such as *Crenarchaeota* (average 12.9%), *Chloroflexi* (average 3.0%), *Planctomycetota* (average 1.3%), and *Acidobacteriota* (average 0.7%), contributing to a more diverse community composition. High-pressure oligotrophic conditions (H51 group) resulted in a microbial community composition and relative abundance closely resembling the original *in situ* community. However, after sub-cultivation, significant shifts were observed, with *Proteobacteria* becoming the most dominant populations, which accounted over 63% on average. Absolute quantification results showed that microbial abundance was highest under atmospheric pressure, particularly in nutrient-rich conditions (A21), and further increased after sub-cultivation under oligotrophic conditions (Figure 3B). In contrast, microbial abundance was lowest under high-pressure cultivation, especially under oligotrophic conditions.

At the genus level, microbial growth under different cultivation conditions was evaluated using growth fold (logarithmic ratio of post-cultivation biomass to original biomass) for the top 15 genera (Figure 3C). The results showed that the biomass of these 15 genera differed significantly from that in the original habitat (Wilcoxon signed-rank test, *p* < 0.05). Most genera exhibited higher growth rate under atmospheric pressure enrichment, while fewer genera showed similar increases under high-pressure enrichment. Genera such as *Pseudomonas* (average relative abundance of 20.4%), *Shewanella* (average 11.4%), *Idiomarina* (average 11.1%), *Brevundimonas* (average 6.7%), and *Halomonas* (average 3.8%) showed significant growth rate under both atmospheric and high-pressure conditions, particularly during the first enrichment under atmospheric pressure and the second enrichment under high pressure. Notably, *Nitrospina* (average relative abundance of 0.2%) and *Exiguobacterium* (average relative abundance of 1.1%), belonging to the *Nitrospinota* and *Firmicutes* phyla respectively, exhibited significant growth fold increases exclusively under high-pressure conditions.

We also analyzed the relative abundance of microorganisms associated with specific functions, such as ammonia oxidation, nitrification, sulfur oxidation, and methane production according to previous studies reported (D'Angelo et al., 2023; Sheik et al., 2014; Trouche et al., 2023; Xie et al., 2024). Ammonia-oxidizing archaea (AOA), such as order *Nitrosopumilales*, exhibited low relative abundance under atmospheric pressure and nutrient-rich conditions (< 0.5%), but their abundance was substantially higher under high-pressure conditions, reaching 39.4% during the first round of cultivation (Figure 3D). Nitrifying bacteria (NOB), including order *Nitrospinales* (average relative abundance of 0.07%), order *Nitrospirales* (average relative abundance of 0.04%), and order *Nitrosococcales* (average relative abundance of 0.004%), as well as sulfur-oxidizing bacteria (SAR324 clade), also displayed higher relative abundances under high-pressure conditions (average relative abundance of 0.04%). Methanogens, such as *Methanomassiliicoccales* and *Methanofastidiosales*, were all detected only in trace amounts (0.004%) in the original samples and were not identified under either atmospheric or high-pressure cultivation conditions.

### Changes in microbial co-occurrence patterns under different cultivation conditions

**Figure 4:**
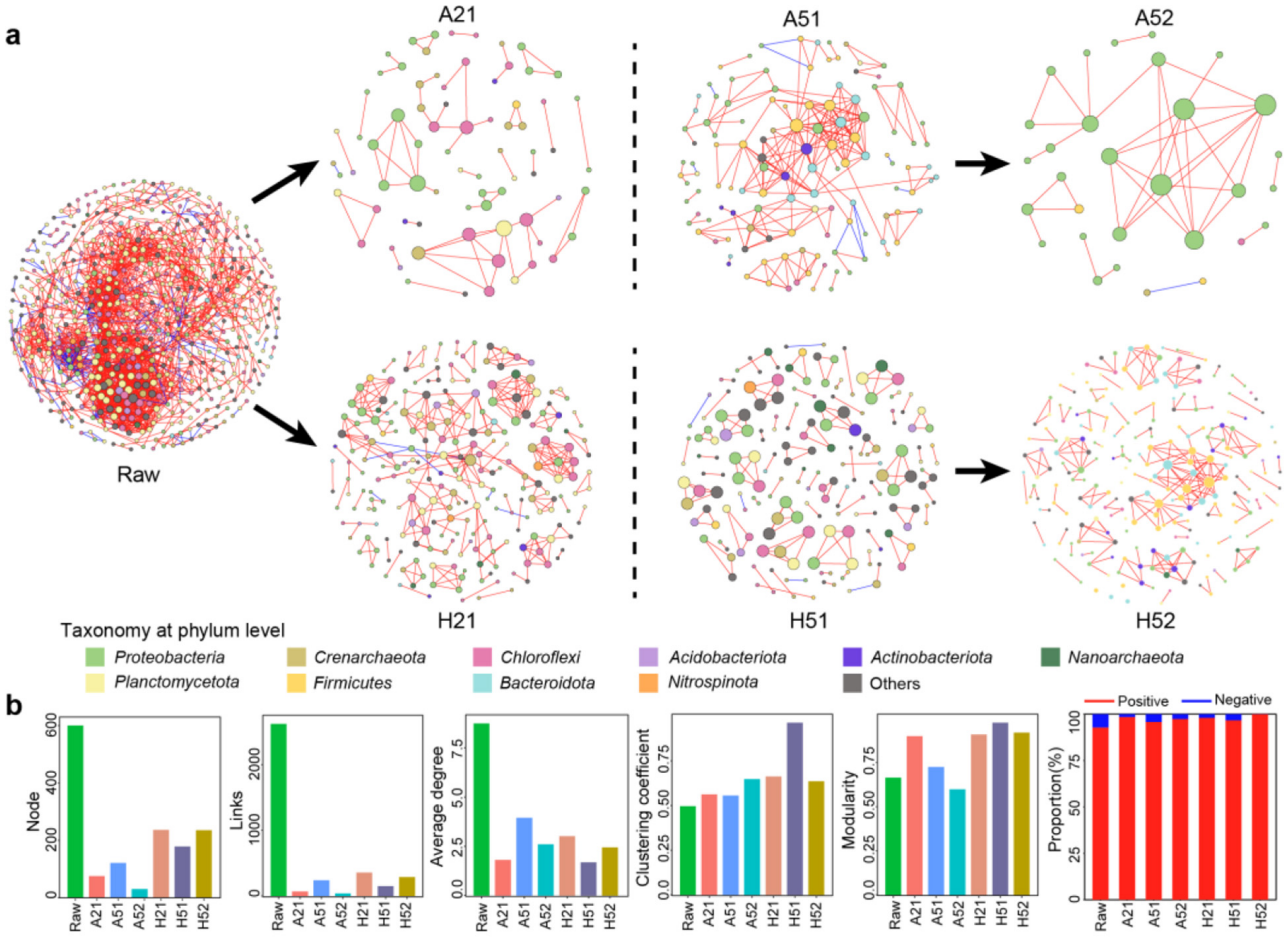
Microbial Co-occurrence Network Analysis under Different Cultivation Conditions. **(a)** Comparison of microbial co-occurrence networks under different cultivation conditions. **(b)** Comparison of the topological characteristics of microbial co-occurrence networks under different cultivation conditions.

Microbial co-occurrence network analysis revealed significant differences in the coexistence patterns of microorganisms across different cultivation conditions (Figure 4A). The original sediment microorganisms exhibited the most complex co-occurrence network, consisting of 600 nodes connected by 2,622 edges. The primary microbial groups in this network included *Planctomycetota, Chloroflexi, Proteobacteria, Crenarchaeo*ta, and *Acidobacteriota* (Supplementary Figure S5). Under atmospheric pressure cultivation conditions, the microbial co-occurrence network was relatively simple, comprising 30–121 nodes, and was predominantly dominated by *Proteobacteria*, particularly after oligotrophic sub-culturing (A52). In contrast, high-pressure cultivation produced more complex co-occurrence networks with 178–236 nodes. The primary microbial groups in high-pressure networks included *Proteobacteria, Planctomycetota, Chloroflexi*, and *Crenarchaeota*.

A comparison of the topological characteristics of these networks demonstrated that high-pressure cultivation resulted in higher node and edge counts, clustering coefficients, and modularity compared to atmospheric pressure cultivation (Figure 4B). Across all conditions, microbial interactions were predominantly positive (over 95%), suggesting that these communities tended to form symbiotic and interdependent relationships rather than competitive ones. Node degree (representing the importance of OTUs within the network) further highlighted differences in microbial network complexity. In the native sediment environment, the top 10 most connected species showed greater phylum-level diversity, encompassing five phyla and two unidentified phyla (Supplementary Table S5). These highly connected species exhibited high connectivity scores (over 37), reflecting complex interrelationships among microorganisms under native conditions. Under atmospheric pressure cultivation, *Proteobacteria* dominated, but node connectivity was relatively low, especially after sub-culturing. High-pressure cultivation, particularly during initial oligotrophic conditions, yielded microbial species with increased diversity among highly connected nodes, but overall connectivity remained low. After sub-culturing under high-pressure conditions, connectivity increased, while species diversity decreased. These findings demonstrate how cultivation conditions, particularly pressure and nutrient availability, influence microbial co-occurrence patterns and network topology, with high-pressure cultivation fostering more complex and interconnected microbial relationships. This highlights the critical role of high-pressure conditions in mimicking native environments and shaping microbial community dynamics.

### Microbial community assembly process under different cultivation conditions

**Figure 5:**
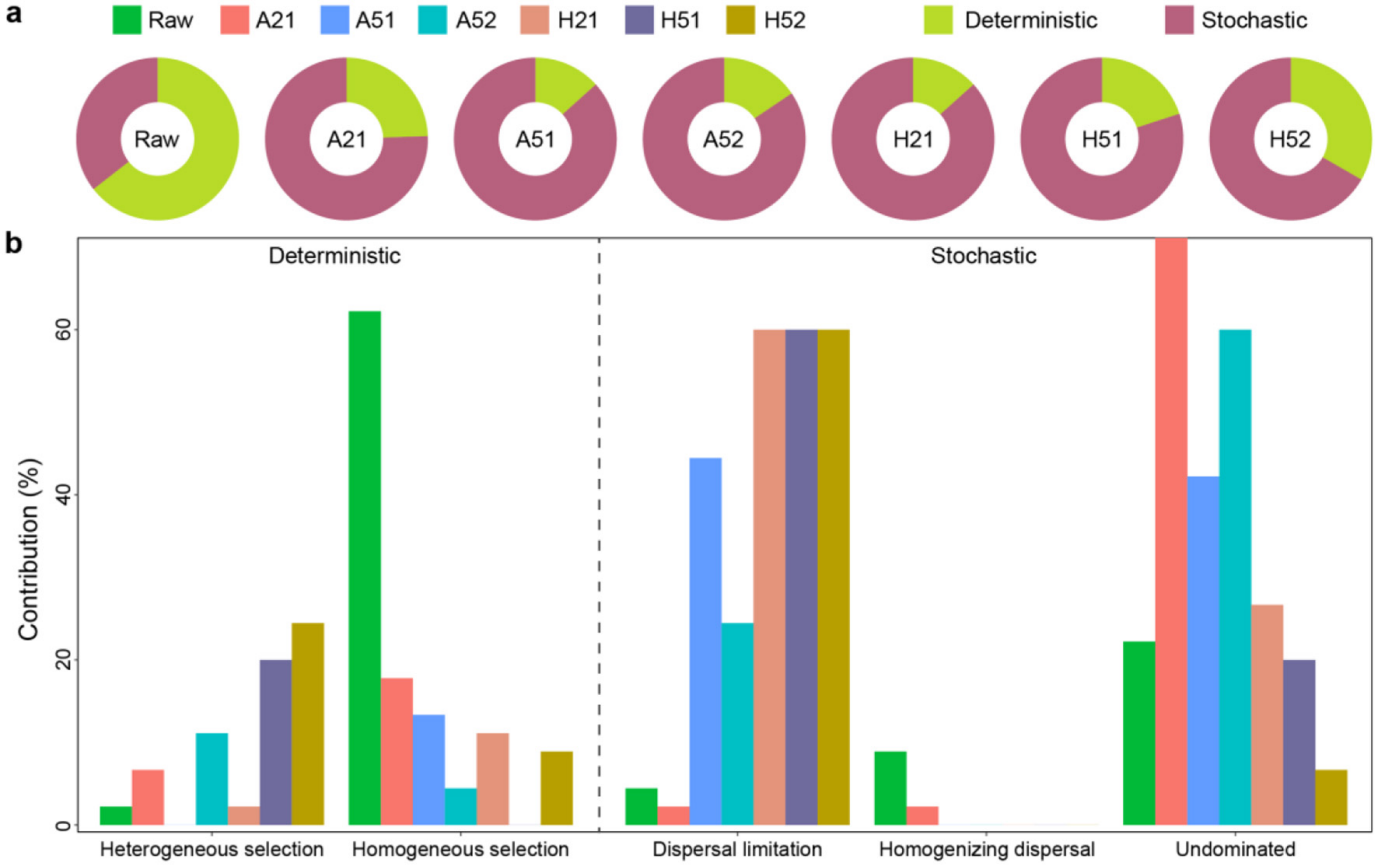
Community assembly processes under different culture conditions. **(a)** Relative proportions of ecological processes under different culture strategies. **(b)** The contribution of community assembly is governed by homogenizing and differentiating processes. Deterministic process = Homogeneous selection + Heterogenous section; Stochastic process = Dispersal limitation + Homogenizing dispersal. Undominated processes include weak selection, weak dispersal, diversification and drift.

The microbial community assembly mechanisms underwent significant transformations under different cultivation conditions compared to the *in situ* microbial communities. In the original sediment environment, community assembly was predominantly governed by deterministic processes (64.4%, Figure 5A), primarily driven by homogeneous selection (62.2%, Figure 5B). Under both atmospheric and high-pressure cultivation conditions, the community assembly process was mainly dominated by stochastic processes (66.7–86.7%). However, there were notable differences: under atmospheric nutrient-rich conditions, the process was primarily undominated (71.1%), while under atmospheric nutrient-poor conditions, stochastic processes were further intensified, and dispersal limitation processes significantly increased. Under high-pressure cultivation conditions, the dispersal limitation process contributed equally to the assembly process under both nutrient-rich and nutrient-poor conditions (60% in both cases). However, under nutrient-poor high-pressure conditions, deterministic processes became more pronounced, particularly after sub-culturing, with an increase in heterogeneous selection. Overall, under nutrient-rich conditions, the heterogeneous selection process became more prominent, while the homogeneous selection process decreased correspondingly. The homogenizing dispersal process was almost entirely absent across all conditions. These findings highlight how nutrient availability and cultivation pressure influence the balance between deterministic and stochastic processes in shaping microbial community assembly. The intensification of deterministic processes under nutrient-poor, high-pressure conditions suggests a strong environmental filtering effect, particularly for sub-cultured communities. This aligns with the natural adaptation of deep-sea microorganisms to extreme environmental conditions, where selective pressures drive the assembly of stable and specialized microbial communities.

## Discussion

### Microbial diversity and community dynamics

Most prokaryotes on Earth reside in the deep ocean biosphere under high hydrostatic pressure (Jebbar et al., 2015), while traditional cultivation methods are inadequate for most deep-sea microorganisms (Liu et al., 2017; Zhang et al., 2019), as these methods fail to meet the distinct pressure requirements of piezophilic (pressure-loving), or piezosensitive (pressure-sensitive) organisms (Amano et al., 2022; Mota et al., 2013). Our findings highlight this limitation, as atmospheric pressure cultivation led to a loss of over 82% of microbial diversity, whereas high-pressure cultivation significantly increased microbial diversity (Figures 2A, B and Supplementary Figure S2), demonstrating that high-pressure cultivation, particularly under oligotrophic conditions (H51). These results indicated that most microorganisms in deep-sea sediments are challenging to culture using traditional methods due to their adaptation to high-pressure, which render them unable to survive under atmospheric pressure (Kato et al., 1998). High-pressure oligotrophic conditions better simulate the natural deep-sea environment, providing suitable conditions for the survival of deep-sea microorganisms and substantially increasing the diversity of culturable microbes (Kato et al., 1998). Conversely, under conventional cultivation, decompression often leads to cell dormancy or death, resulting in significant diversity loss (Cario et al., 2022). For example, MT41, the first obligately barophilic strain isolated from the Mariana Trench, exhibited the fastest grow rate at 100 MPa but cannot grow at pressures below 50 MPa (Yayanos et al., 1981).

Our results also revealed that the microbial community structure and composition obtained under high-pressure, particularly under oligotrophic conditions, closely resembled those of the raw sediments (Figures2C, 3A). This similarity likely arises because oligotrophic conditions align with the nutrient-poor marine ecosystems, enabling the survival of both piezophilic and piezotolerant microorganisms (Bartelme et al., 2020; Overmann et al., 2017). However, despite the structural and compositional resemblance (Figures2C, 3A), the biomass under high-pressure was significantly lower than that under atmospheric pressure cultivation or *in situ* samples (Figure 3B). This is because high-pressure conditions are known to reduce microbial activity (Amano et al., 2022) and slow growth rates (Cario et al., 2022; Kato et al., 2018), which making it difficult for them to achieve high abundance in a short period of time (Marietou and Bartlett, 2014). For instance, *Pseudomonas bathycetes*, the first strain isolated from a trench, has a generation time of 33 days under high-pressure (Kato et al., 1998), with a growth rate only 1/10,000th of that of microorganisms growing at atmospheric pressure. Similarly, SAR11 clade bacteria, among the most abundant and ubiquitous bacteria in the ocean, require up to 24 weeks of incubation to be enriched (Song et al., 2009). Moreover, high-pressure conditions negatively impact nutrient utilization capacity (Pope et al., 1975), protein structure and activity (Jebbar et al., 2015), and respiratory chain efficiency (Chikuma et al., 2007) collectively impeding rapid biomass accumulation.

Under atmospheric pressure, fast-growing microbial groups such as *Pseudomonas, Shewanella*, and *Idiomarina* (from the *Proteobacteria* phylum) rapidly proliferate once the pressure constraint is removed, allowing them to dominate the community. This results in simplified community composition and higher biomass (Figures 3A–C). Some pressure-tolerant microbes, such as *Shewanella*, can grow under both atmospheric and high-pressure conditions (Figure 3C), likely due to pressure-regulating mechanisms (Li et al., 1998), such as producing eicosapentaenoic acid (EPA) or long-chain polyunsaturated fatty acids (PUFAs) to maintain membrane fluidity under high-pressure, low-temperature conditions (DeLong and Yayanos, 1986; Kato et al., 1998; Nogi et al., 1998). Interestingly, subculturing led to an increase in microbial abundance (Figure 3B). After initial adaptation during the first round of cultivation, microorganisms exhibited higher activity, enabling further growth in fresh media. However, subculturing under high-pressure conditions caused significant shifts in microbial community composition and relative abundance. Specifically, the dominance shifted from *Crenarchaeota* to *Proteobacteria* (Figure 3A). This shift is likely due to competitive microbes within the pre-established community rapidly utilize resources, outcompeting slower-growing species and achieving higher relative abundance. At the phylum level, *Crenarchaeota, Chloroflex*i, *Planctomycetota, Acidobacteriota*, and *Nitrospinot*a were significantly enriched only under high-pressure conditions, indicating their likely tolerance to high-pressure environments. In contrast, *Proteobacteria* and *Firmicute*s were enriched under both atmospheric and high-pressure conditions, demonstrating their pressure tolerance. Notably, *Planctomycetota* and *Nanoarchaeota* showed higher abundance under high-pressure oligotrophic conditions, likely due to their efficient nutrient utilization strategies (Dai et al., 2022). For instance, *Planctomycetota* are known for their high-affinity substrate systems, making them well-suited for oligotrophic environments (Overmann et al., 2017). These findings underscored the importance of high-pressure and oligotrophic cultivation conditions in preserving deep-sea microbial diversity and community dynamics, providing critical insights into the adaptations of microorganisms in extreme environments. Additionally, they highlighted the limitations of atmospheric pressure cultivation, which tends to favor fast-growing, opportunistic species over specialized deep-sea microbes. By better replicating natural conditions, high-pressure cultivation techniques represent a valuable tool for advancing our understanding of deep-sea microbiology and ecological function.

### Shifts in microbial coexistence patterns and community assembly processes

Co-occurrence networks are a powerful tool for analyzing potential interactions among community members, identifying microbial social modules, and estimating the ecological significance of specific operational taxonomic units (OTUs) within a community (Faust and Raes, 2012; Xian et al., 2020). In this study, we observed that under high-pressure oligotrophic conditions, microbial communities formed more complex networks with higher modularity and were dominated by positive correlations (Figure 4), despite exhibiting relatively low growth rates. This indicated that in high-pressure, nutrient-limited environments, microorganisms tend to establish cooperative relationships (e.g., symbiosis and mutualism) to sustain community stability and diversity. Such conditions promote a more modular (compartmentalized) network organization, which has been linked to increased robustness/persistence and can facilitate niche partitioning and stable coexistence in ecological communities (Grilli et al., 2016; Lurgi et al., 2019; Sheykhali et al., 2020). This behavior likely reflects the need to maximize resource use under the combined stresses of high pressure and nutrient scarcity (Zengler and Zaramela, 2018). Certain microbes exhibited enhanced abilities for organic matter degradation and nitrogen cycling under high-pressure conditions (Yang et al., 2024), while others adopt distinct survival strategies and spatial distribution patterns depending on nutrient availability (Liu et al., 2022a; Zhao et al., 2024). High pressure may suppress individual microbial activity, encouraging metabolic interdependence for organic matter degradation (Amano et al., 2022; Glud and Schauberger, 2024; Stief et al., 2023). This interdependence leads to metabolic complementarity, providing essential nutrients and energy to other microorganisms and fostering complex microbial interactions (Dai et al., 2022; Huang et al., 2024; Peng et al., 2024). In native sediment samples, *Planctomycetota, Chloroflexi, Crenarchaeota*, and *Proteobacteria* were dominant in the co-occurrence network. These groups are key drivers of organic matter degradation and carbon-nitrogen cycling (Chen et al., 2021; Dong et al., 2022; Yang et al., 2024). Under high-pressure stress, these taxa likely form intricate “social networks” to achieve complementary survival strategies (Ghoul and Mitri, 2016), such as forming cross-feeding and close interactions with other community members or hosts become essential for their survival in such extreme environments (Janssen, 2003; Peng et al., 2024; Zengler and Zaramela, 2018). These findings highlighted how high-pressure and nutrient-limited conditions drive the formation of co-occurrence patterns (Dai et al., 2022), enhancing the stability and functionality of microbes in extreme environments.

Pressure imposes significant selective forces on microbial community assembly (Jebbar et al., 2015), however, understanding how pressure influences deep-sea microbial communities and their structures remains a critical topic for further investigation. Compared to community assembly dominated by stochastic processes under atmospheric pressure, deterministic processes were significantly enhanced under high-pressure conditions, particularly through the increasing role of heterogeneous selection. This suggested a shift in microbial community assembly from stochastic to deterministic processes as cultivation conditions change. Our findings aligned with observations that microbial communities in natural habitats are primarily shaped by deterministic processes, reflecting evolutionary adaptations to environmental pressures (Kohl, 2020). Interestingly, although A51 and A52 were expected to impose stronger environmental filtering than A21 due to lower nutrient supply, the inferred contribution of deterministic processes was lower than in A21, which may result from several non-mutually exclusive factors. First, if the A52 medium reduced niche differentiation (e.g., because all taxa experienced similarly low nutrient availability), growth could slow across taxa and competitive differences may weaken, thereby increasing the relative importance of stochastic colonization and ecological drift. Second, early enrichment at 1 atm may amplify priority effects, whereby small differences in the initial inoculum composition and growth timing are magnified and propagated into divergent final community states, increasing the apparent stochastic component. In addition, atmospheric-pressure incubations often favor fast-growing, functionally similar copiotrophs, leading to convergence in trait space that may not carry a strong phylogenetic signal, thereby weakening selection signals detected by phylogeny-based null models. Finally, differences in biomass yield and sequencing depth among groups can affect the detection of rare taxa and alter beta-diversity structure, which may in turn influence the partitioning of assembly processes. Therefore, we interpret the A52-vs.-A21 contrast as reflecting interacting effects of resource regime, early colonization dynamics, and the limited ability of atmospheric-pressure enrichments to maintain *in situ*-like selective constraints. Microbial community formation under high-pressure oligotrophic conditions appears to be driven by species' adaptive responses to environmental selection rather than random colonization (Huang et al., 2023a; Laugier, 2023). Dispersal limitation also plays a critical role in community assembly under high-pressure conditions (Figure 5B), that probably because environmental pressures may restrict microbial distribution and reproduction, resulting in a more concentrated and stable community composition dominated by species with strong survival capabilities (Sebastián et al., 2021). The combination of dispersal limitation and heterogeneous selection likely stabilizes community composition and structure under extreme environmental pressures. These results suggested that microbial community assembly processes under high-pressure oligotrophic conditions closely resemble those in natural deep-sea environments. In these ecosystems, extreme pressures drive adaptive evolution, enabling deep-sea microbial communities to sustain high diversity and intricate interrelationships. Simulating high-pressure oligotrophic conditions not only facilitated the enrichment of deep-sea microorganisms but also provided a framework for understanding the formation and evolutionary mechanisms of microbial communities in extreme environments. These insights were critical for advancing our knowledge of deep-sea microbiology and its ecological and biogeochemical significance.

## Conclusion

This study examined the impact of hydrostatic pressure and nutrient availability on the diversity, dynamics, co-occurrence patterns, and assembly processes of deep-sea microorganisms from Yap Trench sediments. Our findings demonstrate that cultivation conditions strongly shape microbial alpha diversity and community composition. High-pressure, oligotrophic incubations preserved higher diversity and yielded community structures more similar to the *in situ* microbiota. Microbial communities under high pressure formed more complex co-occurrence networks with higher modularity and predominantly positive associations, consistent with enhanced niche partitioning and more stable coexistence patterns in extreme environments. Community assembly analyses further indicated an increased contribution of deterministic processes under high pressure, with heterogeneous selection and dispersal limitation playing prominent roles, highlighting the importance of reproducing key *in situ* constraints to access otherwise recalcitrant taxa. Beyond pressure and oligotrophy, future deep-sea cultivation may be further improved by incorporating near-*in situ* temperature and extended incubation, realistic redox and electron-acceptor regimes, naturalistic substrate forms and low-concentration delivery, mineral matrices, appropriate salinity/pH and trace growth factors, and co-culture or diffusion-based cultivation strategies. Together, these approaches provide a practical roadmap for enriching diverse deep-sea microbial groups, enabling mechanistic studies of their roles in biogeochemical cycling and expanding microbial resources for biotechnological applications.

## Data Availability

The original contributions presented in the study are included in the article/Supplementary material, further inquiries can be directed to the corresponding author.
