# Supplementary material for: Variations in deep-sea microbial composition and assembly mechanisms under different culture strategies
Source: Front Microbiol. 2026 Mar 18;17:1783610. doi: 10.3389/fmicb.2026.1783610 (PMC13038925; doi:10.3389/fmicb.2026.1783610)
Supplement: Supplementary file 1 [file Data_Sheet_1.docx]

*Supplementary Figures of the article:*

**Variations in deep-sea microbial composition and assembly mechanisms under different culture strategies**

by Yu *et al*

**This additional information contains:**

- - 4 Pages
  - 5 Figures

**
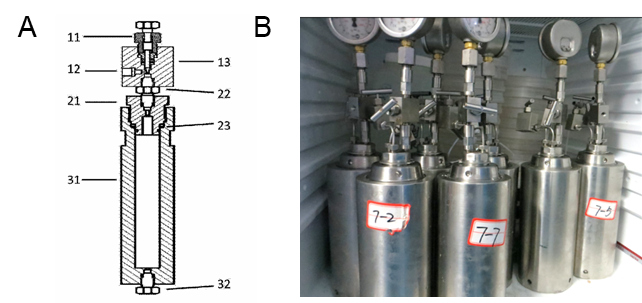
**

Fig. S1. Schematic diagram and photographs of the portable high-pressure cultivation device. (A) Schematic of the portable high-pressure microbial transfer and culturing device, consisting of a pressurization interface unit (1), a high-pressure sealing unit (2), and a high-pressure cultivation unit (3). The pressurization interface unit (1) is connected to the high-pressure cultivation unit (3) via the high-pressure sealing unit (2). The pressurization interface unit (1) includes the following components: a handleless high-pressure needle valve (11) connected to the pressurization interface; a pressurization interface (12) with an embedded threaded standard connector, which links the external high-pressure source to the handleless high-pressure needle valve and communicates with the cultivation unit through the sealing unit; and a three-way connecting block (13), in which the first channel connects to the handleless high-pressure needle valve, the second channel connects to the pressurization interface, and the third channel connects to the high-pressure sealing unit. The high-pressure sealing unit (2) includes: a reactor lid (21) that is threaded and sealed onto the top of the high-pressure cultivation unit (3), with an internal through-hole allowing liquid to pass; a hollow connector (22), one end connected to the pressurization interface unit (1) and the other end connected to the reactor lid (21), with an internal channel permitting liquid flow; and a sealing ring (23) embedded in the lower edge of the reactor lid. The high-pressure cultivation unit (3) includes: a reactor body (31) containing the cultivation chamber, with its upper end threaded to the sealing unit; and a bottom opening (32) located at the base of the reactor body (31). (B) Photograph of the device placed in a refrigerator (2 °C) for microbial enrichment cultivation.


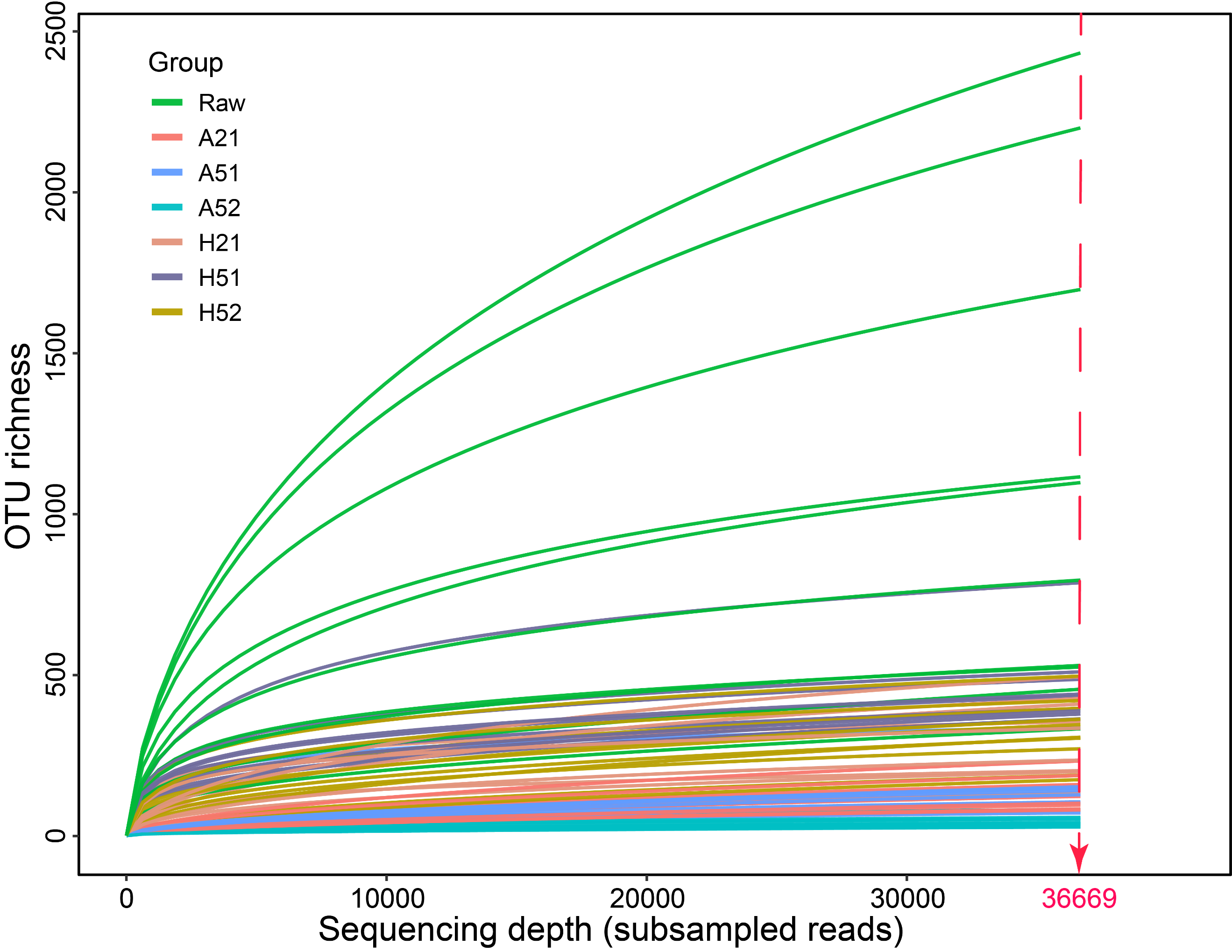


**Fig. S2.** The rarefaction curve of sequencing depth versus species diversity.


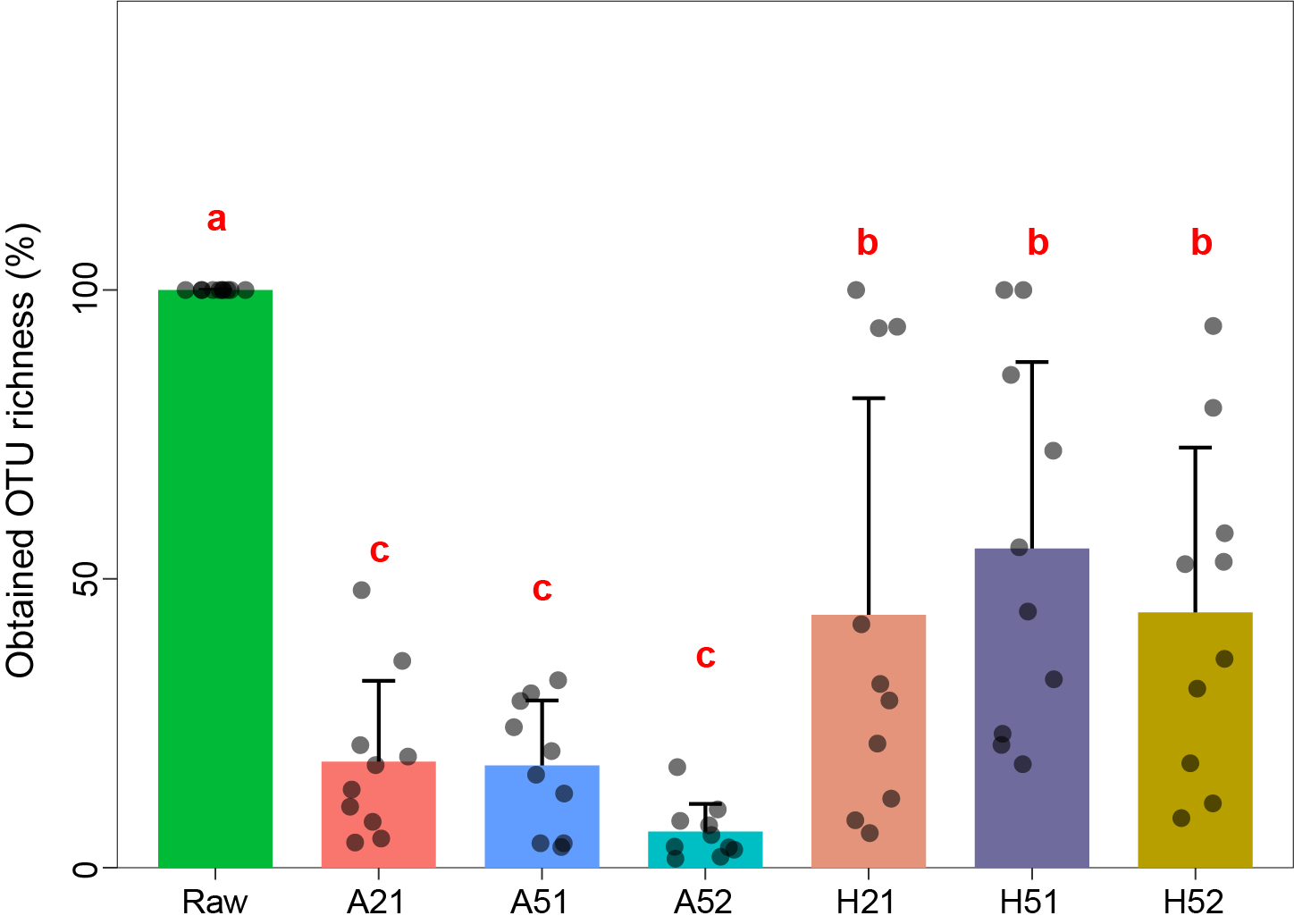


**Fig. S3.** Proportions of microbial OTUs obtained under different cultivation conditions compared to raw samples. Pairwise comparisons of obtained OTU richness were compared using Dunn’s post hoc with Benjamini–Hochberg correction.


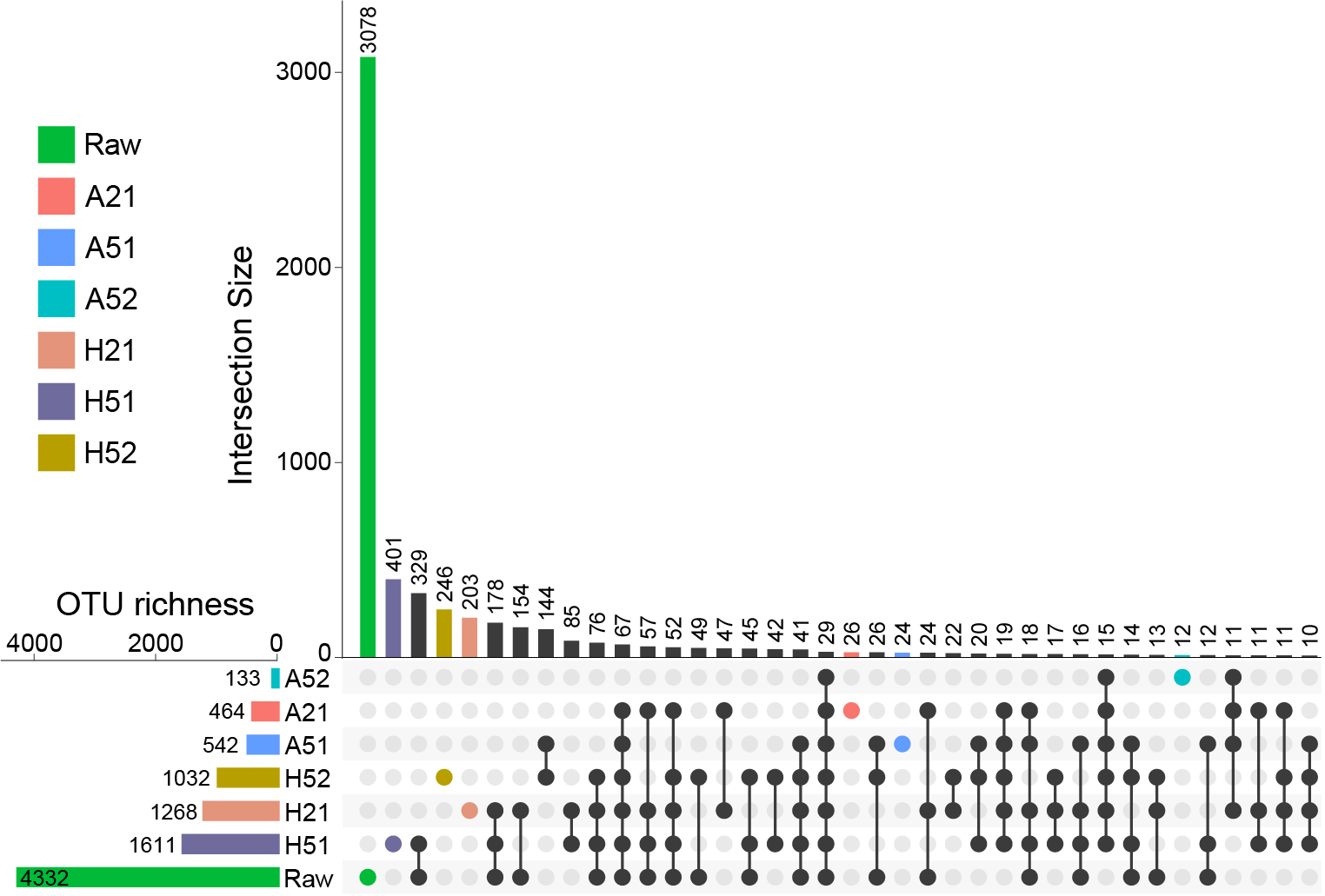


**Fig. S4.** Shared and unique microbial OTU counts under different cultivation conditions.


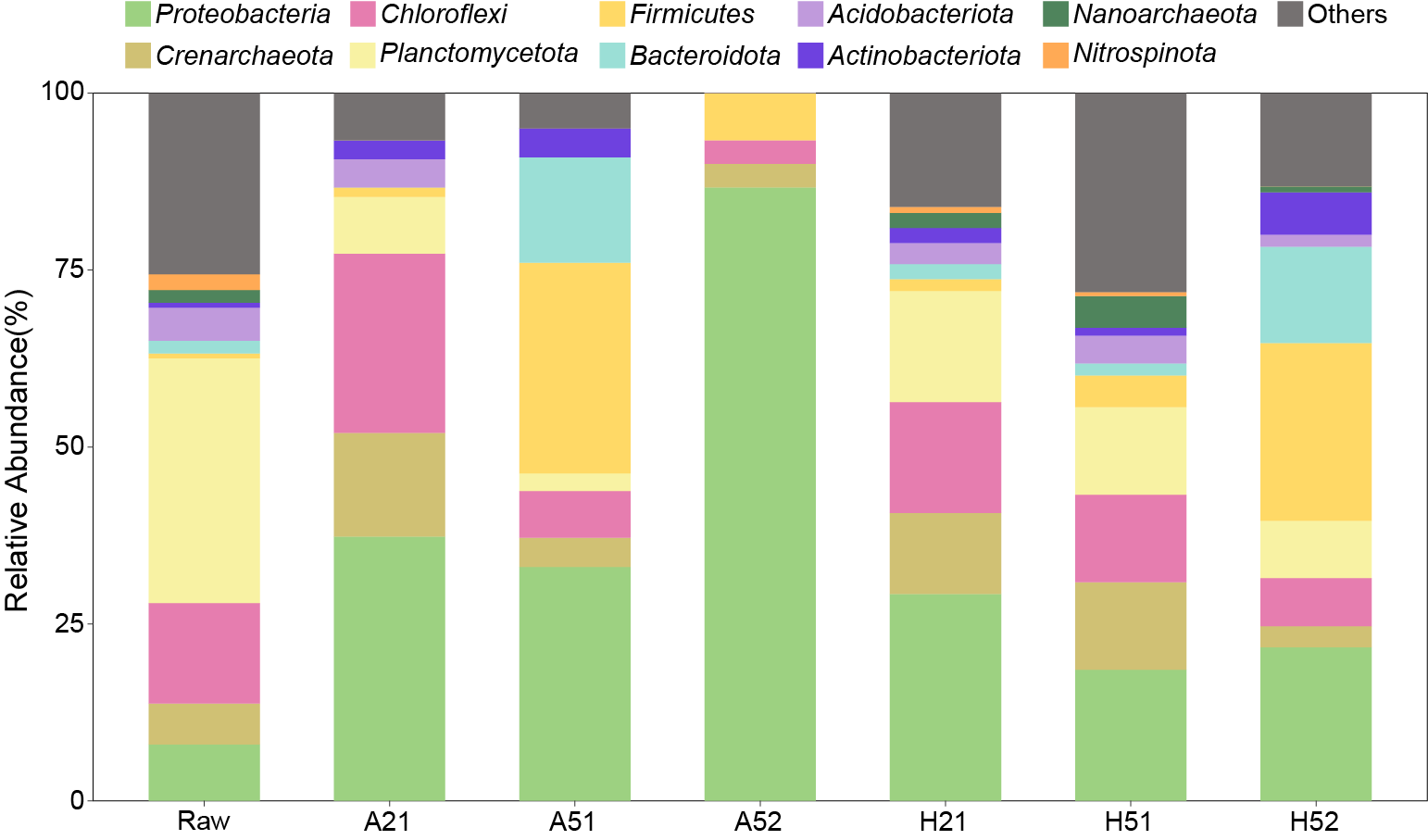


**Fig. S5.** Microbial composition within co-occurrence networks under different cultivation conditions.
